# Ginger and Testosterone

**DOI:** 10.3390/biom8040119

**Published:** 2018-10-22

**Authors:** Saleem Ali Banihani

**Affiliations:** Department of Medical Laboratory Sciences, Jordan University of Science and Technology, Irbid 22110, Jordan; sabanihani@just.edu.jo; Tel.: +962-2-7201000

**Keywords:** ginger, *Zingiber officinale*, gingerol, testosterone

## Abstract

Enhancing and protecting testosterone production is one target for many scientists because of its crucial role as a primary sex hormone in males. Several in vivo trials have utilized different dietary supplements and medicinal plants to enhance testosterone production in males. Since 1991, various in-vivo, as well as basic research studies, have discovered a link between ginger (*Zingiber officinale*) and testosterone. However, such a link has not yet been collectively reviewed. This review systematically discusses and summarizes the effect of ginger and ginger extracts on testosterone. To achieve this contribution, we searched the PubMed, Scopus, and Web of Science databases for English language articles (full texts or abstracts) from November 1991 through August 2018 using the keywords “ginger” and “*Zingiber officinale*” versus “testosterone”. Additionally, the references from related published articles were also reviewed, only if relevant. In conclusion, the mainstream of research that links ginger to testosterone demonstrated that ginger supplementation, particularly in oxidative stress conditions, enhances testosterone production in males. The mechanisms by which this occurs mainly by enhancing luteinizing hormone (LH) production, increasing the level of cholesterol in the testes, reducing oxidative stress and lipid peroxidation in the testes, enhancing the activity of the antioxidant enzymes, normalizing blood glucose, increasing blood flow in the testes, increasing testicular weight, and recycling testosterone receptors. However, the effect of ginger on testosterone is not yet confirmed in humans. Therefore, clinical studies in this context of research are imperative.

## 1. Introduction

Ginger (*Zingiber officinale*) is a flowering medicinal plant whose root, or rhizome (plant stem) is commonly used as a spice [1]. Additionally, it is widely used in folk medicine because of its many health benefits in various diseases, including chronic diseases such as diabetes [2,3], cancer [4,5,6], ulcer [7,8], Alzheimer [9], cardiovascular disease [10,11], and depression [12]. The beneficial effect of ginger in such diseases is mainly due to its antioxidant [13,14], antimicrobial [15], and anti-inflammatory properties [16].

The sharp fragrance and flavor of fresh ginger root result from certain bioactive volatile-oils (e.g., gingerols, shogaols, and zingerone) that comprise approximately 1–3% of its weight [17]. 6-gingerol is considered as the major pungent and the main bioactive compound in fresh ginger [18]. In addition, ginger contains several antioxidant compounds such as vitamin C, vitamin E, beta-carotene, lutein, lycopene, quercetin, genistein, and tannin [19]. Moreover, ginger contains essential elements such as manganese, copper, selenium, and zinc [19]. Besides, ginger has been found to contain low amounts of toxic elements such as cadmium, lead, and nickel [20].

Enhancing testosterone production in human males and other male species is still the ultimate goal for many scientists in the field. Such an intention is due to the crucial function of testosterone as the major sex hormone in males [21]. Testosterone plays an important role in developing male reproductive organs and promoting other sexual characteristics such as the growth of body hair and increased bone and muscle mass [22]. In addition, testosterone is involved in general health and well-being [23]. Insufficient levels of testosterone in men are linked with a wide range of disorders/diseases such as infertility [24], diabetes [25], osteoporosis [26], and bone loss [27]. Therefore, several in vivo studies have investigated various dietary supplements as well as medicinal plants on the level of testosterone in males.

Since the beginning of 1991, various in vivo and basic research studies have found a link between ginger and testosterone. However, whether systematically or narratively, such a link has not yet been collectively discussed. Here, we systematically review the effect of ginger on the testosterone level in males. To achieve this contribution, we searched the PubMed, Web of Science, and Scopus databases for English language articles (published in full texts or only abstracts) from November 1991 through August 2018 using the keywords “ginger” and “*Zingiber officinale*” versus “testosterone”. Additionally, the references from related published papers were also reviewed, only if relevant.

## 2. Effect of Ginger on Testosterone

Until now, almost all studies that have found a direct link between ginger and testosterone were in vivo studies. Table 1 summarizes the direct studies conducted on ginger and its extracts or derived compounds and their reported effects on serum testosterone. Almost all of these studies were conducted on diabetic rat models. As shown in the table, diabetic and hypertensive rat models supplemented with ginger and its extracts had a higher serum testosterone level compared to controls. While ginger-derived compounds (zingerone, geraniol, and 6-gingerol), when taken separately, did not affect serum testosterone level in diabetic rats.

The other set of studies that reported the direct link between ginger and testosterone were reproductive toxicity studies. Table 2 summarizes all of these studies, which were conducted on ginger and its derived compounds, and their reported protective effects on serum testosterone level. Also, to date, almost all of the reproductive toxicity studies are rodent studies.

In general, reproductive toxicity studies were designed to investigate the influence of ginger in ameliorating the testosterone level mainly in toxican-induced male rats. These toxicans were, most of the time, chemical compounds (e.g., aluminum chloride, sodium metabisulfite), metals (e.g., lead), or drugs. Examples of the drugs used in this context, which induce reproductive toxicity, are lamotrigine (antiepileptic drug), cyclophosphamide (anti-cancer drug), busulfan (anti-cancer drug), and carbendazim (fungicide drug).

As a general trend, the studied toxicans reduced the level of testosterone in the experimental animals, and ginger supplementation counteracted this reduction. The ginger doses that are used in the reproductive toxicity studies vary from ~40 to ~600 mg daily, and the duration of supplementation ranged from ~2 to ~8 weeks.

## 3. Mechanistic Studies

In men, more than 95% of testosterone is produced by the testis, while the remainder is produced by other organs, mainly the adrenal glands [22]. The testis contains two main types of cells: Leydig cells and Sertoli cells. The synthesis of testosterone occurs in the Leydig cells, while the Sertoli cells utilize the produced testosterone for spermatogenesis. Chemically, similar to other androgens, testosterone is derived from cholesterol [40]. The in vivo system study conducted by Kamtchouing et al. in 2002 showed that supplementation of ginger at 600 mg kg^−1^ for eight days increases the level of testicular cholesterol [28], which could be a seminal factor behind the increased testosterone production after ginger administration.

Ginger root is rich with several potent antioxidant compounds such as gingerols, zingerone, zingiberene, glucosides-6-gingerdiol, flavonoids, and volatile oils [17]. These antioxidants protect both the reproductive organs from oxidative stress, an imbalance between prooxidants (reactive oxygen species such as superoxide ion, hydroxyl radical, and hydrogen peroxide) and antioxidants to the favor of the former [41], and lipid peroxidation. Ginger roots were found to enhance the activity of certain antioxidant enzymes such as superoxide dismutase (SOD), catalase (CAT), and glutathione peroxidase (GPx) in different male reproductive organs such as testis, prostate, and epididymis [32]. In addition, ginger has been found to attenuate the cell damage markers such as aspartate aminotransferase (AST), alanine aminotransferase (ALT), alkaline phosphatase (ALP), and lactate dehydrogenase (LDH) in the testis [32]. Accordingly, the reduction in oxidative stress and enhancement of the antioxidant defense mechanism against prooxidants in testes cells may enhance the biosynthesis of testosterone.

The evidence above explains why the majority of studies that demonstrate the effect of ginger on testosterone were conducted on diabetic rat models. Various studies have shown that free radical generation, and hence the level of cellular oxidative stress, in diabetic conditions is higher than the normal conditions [42,43,44], which may negatively affect cellular function and cellular biosynthesis [45].

Moreover, in fact, normal Leydig cell function is affected primarily by luteinizing hormone (LH) [46], which plays a major role in testosterone synthesis. It has been shown that hyperglycemia induces abnormal changes in the Leydig cells leading to a change in the pituitary-testicular axis, including a decrease in LH level, and this subsequently decreases the synthesis of testosterone [47]. Ginger has been found to increase LH production in diabetic rat models [46], which consequently improves the synthesis of testosterone.

Further, in hypertensive rats, the level of reactive oxygen species and thiobarbituric acid reactive substances (TBARS), a byproduct of lipid peroxidation, is increased in testes and epididymis [30]. Excessive formation of TBARS may cause overutilization of glutathione S-transferase and glutathione (GSH), a potent synthetic antioxidant. The decrease in GSH level reduces the detoxification process for prooxidants in the testes, which negatively affects the testosterone production [30]. It has been shown that dietary supplementation of ginger roots prevented the decrease in glutathione S-transferase and GSH level thereby resulting in a reduction in prooxidants, which may subsequently enhance the synthesis of testosterone [30].

Furthermore, in diabetic conditions, other than its potential antioxidant effects in the testes, ginger roots have been found to have a direct effect on blood glucose [32]. The study conducted on alloxan-induced diabetic rats by Ghlissi et al. in 2013 showed that 1.5 g of fresh ginger per 15 g of rat diet, for 30 days, significantly reduced the level of blood glucose [32]. In addition, Streptozotocin-induced diabetic rats treated orally with 500 mg kg^−1^ of ginger extract daily had lower glycated hemoglobin (HbA1_C_) compared to a control group [48]. In fact, several studies have introduced the antihyperglycemic effect of dietary ginger on blood glucose [49,50,51]. On the other hand, studies have shown that, in diabetic conditions, the amelioration of blood glucose enhances testosterone production [52,53]. Accordingly, ginger potentially increases testosterone production in hyperglycemic conditions by normalizing blood glucose level.

It has been shown that the reduction in blood flow to the testes decreases the production of testosterone, which may lead to hypo-spermatogenesis [54,55]. The nitric oxide-cyclic guanosine monophosphate (NO-cGMP) pathway has been found to play a major role in male sexual function by inducing the production of NO, which is known as a potent vasodilator [56,57]. It is well-known that the vasodilation effect of NO increased blood flow in the blood vessels [58,59]. Ghareib et al. concluded that 6-gingerol, which is a potent bioactive compound in ginger, is able to stimulate cGMP and enhance the production of NO [60]. Accordingly, dietary ginger root may enhance testosterone production by boosting the production of NO and increasing the blood flow in the testis.

Testicular weight is an important anatomical indicator of the fertilization ability of males [61,62]. Various studies have measured testicular weight as a marker for reproductive capability following dietary supplements. Testicular weight is directly proportional to the level of testosterone produced. Male rats supplemented with ginger or ginger extracts, for at least one week, had a higher testicular weight compared with a control group; Hence, they had a higher testosterone level, given that it is well-known that testicular weight is proportional directly with the level of testosterone produced [29,32].

Nutritionally, ginger contains amounts of vital nutrients that may enhance testosterone production such as manganese. The study conducted by Koch et al. (2017) indicated that ginger roots contain high amounts of manganese [20]. It has been shown that manganese supplementation stimulated LH secretion in male rats, which may consequently enhance testosterone production [63].

This study has some limitations. Thus far, all studies that have found a direct link between ginger or its derived compounds and testosterone were in vivo system studies, and no human studies were conducted in this specific research context. Therefore, the results presented in this study were reliant on only in vivo system studies.

## 4. Conclusions and Future Perspectives

To date, the mainstream of research linking dietary ginger to testosterone has revealed that ginger or ginger extracts have an impact on testosterone as testosterone production was enhanced upon ginger supplementation.

The mechanisms through which ginger enhances testosterone production are mainly by increasing LH production, increasing the level of cholesterol in testes, reducing oxidative stress and lipid peroxidation in the testes, enhancing the activity of certain antioxidant enzymes, normalizing blood glucose, enhancing nitric oxide production and increasing blood flow in Leydig cells, increasing testicular weight, and recycling testosterone receptors.

However, the effect of ginger or ginger extracts on testosterone is not yet confirmed in humans. Therefore, human studies in this context of research are of great importance.

## Figures and Tables

**Table 1:** A summary of the studies conducted on ginger and its extracts or derived compounds and their reported effects on testosterone.

Affecter	Dose	Duration	Population	Effect on Serum Testosterone	Ref.
Aqueous extract of ginger	600 mg kg^−1^	8 days	Streptozotocin-induced diabetic rats	(+)	[28]
Methanolic *Zingiber officinale* roots	100 and 200 mg kg^−1^	65 days	Alloxan-induced diabetic rats	(+)	[29]
Water extracts of *Zingiber officinale* roots	150 and 300 mg kg^−1^	65 days	Alloxan-induced diabetic rats	(+)	[29]
Ginger	4% of the diet	2 weeks	Hypertensive male rats	(+)	[30]
Zingerone	20 mg kg^−1^	8 weeks	Streptozotocin-induced diabetic rats	(±)	[31]
geraniol	200 mg kg^−1^	8 weeks	Streptozotocin-induced diabetic rats	(±)	[31]
6-gingerol	75 mg kg^−1^	8 weeks	Streptozotocin-induced diabetic rats	(±)	[31]
Fresh ginger roots	1.5 g/15 g of diet	30 days	Alloxan induced male diabetic rats	(+)	[32]

(+) Increase, (−) decrease, (±) no effect.

**Table 2:** A summary of the reproductive toxicity studies conducted on ginger and its derived compounds and their reported protective effects on testosterone level.

Toxican	Population	Effect of Toxican on Testosterone	Ginger Affecter (Mode of Treatment): Dose/Duration	Protective Effect of Ginger on Testosterone	Ref.
Aluminium chloride	Albino Wistar male rats	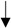	Fresh ginger (Orally): 40 mg kg^−1^ body weight, for 60 days.	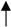	[33]
Lead	Male Sprague Dawley rats	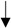	Fresh ginger (Orally): 0.5 and 1 gm kg^−1^ body weight, for 2, 4, and 6 weeks.	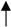	[34]
Lamotrigine	Adult Wistar albino rats	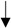	Ginger powder (Orally): 100 mg kg^−1^ daily, for 4 weeks.	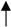	[35]
Cyclophosphamide	Wistar male rats	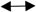	Water ginger extract (Intraperitoneally): 300 or 600 mg daily, for 6 weeks.	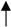	[36]
Busulfan	Male Sprague Dawley rats	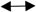	Alcoholic extract of ginger (Orally): 50, 100 and 150 mg kg^−1^ body weight, for 48 days.	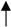	[37]
Carbendazim	Male Sprague Dawley rats	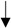	6-Gingerol-rich fraction from ginger (Orally): 50, 100 and 200 mg kg^−1^, for 14 days.	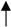	[38]
Sodium metabisulfte	Male Wistar rats	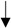	Water ginger extract (Orally): 500 mg kg^−1^ daily, for 28 days.	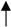	[39]

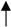
 Increase, 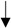 decrease, 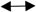 no effect.
